# Intellectual Functioning in Offspring of Parents with Bipolar Disorder: A Review of the Literature

**DOI:** 10.3390/brainsci7110143

**Published:** 2017-10-28

**Authors:** Bonnie Klimes-Dougan, Jake Jeong, Kevin P. Kennedy, Timothy A. Allen

**Affiliations:** 1 Department of Psychology, University of Minnesota, 75 East River Road, 412 Elliot Hall, Minneapolis, MN 55455, USA; jeong132@umn.edu; 2 School of Medicine, University of Chicago, Chicago, IL 60601, USA; kevpkenny@gmail.com; 3 Center for Addiction and Mental Health, Campbell Family Mental Health Research Institute, Toronto, ON M5N 1G3, Canada; timothy.allen@camh.ca

**Keywords:** IQ, intelligence, family risk, bipolar disorder, offspring

## Abstract

Impaired intellectual functioning is an important risk factor for the emergence of severe mental illness. Unlike many other forms of mental disorder however, the association between bipolar disorder and intellectual deficits is unclear. In this narrative review, we examine the current evidence on intellectual functioning in children and adolescents at risk for developing bipolar disorder. The results are based on 18 independent, peer-reviewed publications from 1980 to 2017 that met criteria for this study. The findings yielded no consistent evidence of lower or higher intellectual quotient (IQ) in offspring of parents diagnosed with bipolar disorder. Some tentative evidence was found for lower performance IQ in offspring of bipolar parents as compared to controls. It is recommended that future research examine variability in intellectual functioning and potential moderators. These findings demonstrate the need to examine how intellectual functioning unfolds across development given the potential role of IQ as a marker of vulnerability or resilience in youth at high risk for affective disorders.

## 1. Introduction

Intellectual resources provide numerous advantages for the developing individual. High intelligence is associated with increased self-control, social competence, academic and occupational success, mental health literacy, and longevity [1,2,3,4,5,6,7,8,9]. High intelligence also serves as a protective factor against mental illness, whereas low premorbid intellectual functioning is a risk factor for many forms of serious psychopathology, including severe depression, schizophrenia, and other nonaffective psychoses [1,10,11,12]. Cognitive deficits are over-represented in those suffering from mental disorders [13,14,15] and are typically associated with a worse prognosis [16,17]. Unlike many other forms of mental disorder however, the relationship between bipolar disorder and intellectual deficits is unclear.

In recent years, there has been renewed interest in the cognitive functioning of those with bipolar disorder (BD), a severe and debilitating illness characterized by periods of mood variability, including episodes of mania (as in Bipolar I Disorder) or hypomania (as in Bipolar II Disorder) and depression [18]. In contrast to the more typical pattern of low intellectual functioning co-aggregating with severe mental illnesses, epidemiological studies using population-based cohorts, birth cohorts, and military conscripts have not consistently shown intellectual impairment in those at risk for or diagnosed with bipolar disorder. There is some evidence that a low premorbid intelligence quotient (IQ) is a risk factor for affective disorders in general [19,20]. Several other studies, however, have found no relationship between intellectual functioning and specific risk for BD [12,21,22,23]. Still others have suggested that BD might be associated with either a more mixed profile of premorbid intellectual performance (e.g., low visuospatial reasoning but high arithmetic functioning predicting BD [24]), or even elevated cognitive functioning [5,25]. One recent birth cohort study in England, for example, found that high IQ (particularly high verbal IQ) in childhood was positively associated with manic symptoms in young adulthood [26]. And, in a sample of nearly a million men, Gale and colleagues [27] found that risk for hospitalization due to BD was increased in individuals with the lowest and the highest levels of intelligence when controlling for other psychiatric comorbidities. Moreover, the association between BD and high intelligence appeared to be most prominent for those who scored highest on tasks assessing verbal and technical, as opposed to logical and spatial intelligence. Taken together, current evidence from large-scale studies appears to cast doubt on the notion that high IQ is a protective factor for BD. Nonetheless, results are inconsistent across studies. Methodological concerns may explain some of the inconsistency, as many of these studies are often limited by over-reliance on data from exclusively male samples and heterogeneous approaches to assessing both cognitive functioning and BD. As a result, there is a prominent need for a method of investigating the association between IQ and risk for bipolar disorder that could provide more granular and developmental data than large epidemiological samples.

One approach for accruing such data is to evaluate cognitive functioning in high-risk family samples (typically a first-degree relative of an individual diagnosed with BD) before and during the age of peak-onset of mood disorders. Family risk studies provide a behavioral genetics approach that can be advantageous for considering how heritability and environmental factors form unique challenges to the individual’s ability to adapt. Offspring studies may be particularly useful. More than four decades of research have documented that being born to and raised by a parent struggling with a mood disorder initiates a probabilistic developmental pathway characterized by heightened risk for psychopathology and impairment across several critical domains of functioning (for reviews see [28,29,30,31]. Importantly, having a parent diagnosed with BD is one of the highest risk factors for the emergence of BD later in life. Estimates indicate that offspring of a parent with bipolar disorder (BDO) are 5 to 10 times more likely to develop BD by adulthood than the general population [32]. While the incidence of BD among the general population is approximately one percent, about nine percent of BDO develop the illness [33]. Due to the high rates of psychopathology among youth of parents diagnosed with an affective disorder, and specifically BD, family risk designs provide a unique avenue by which researchers can examine an array of potential risk and protective factors, including the relationship between intellectual functioning and risk for psychopathology. 

Preliminary evidence from the reviews that touch on this topic [34,35,36], is inconclusive, but suggests that lower perceptual processing may be a risk factor for future psychopathology. In this narrative review of the literature, we compile the studies that evaluate intellectual functioning in BDO, including a number of studies that have been recently published on this topic. We pay particular attention to patterns of intellectual functioning in BDO who are assessed in childhood or adolescence, before the age when BD most commonly develops. By examining this literature, we hope to determine whether intellectual functioning is impaired or enhanced in BDO.

## 2. Material and Methods

We searched Medline and PsychInfo using the terms ([“bipolar” or “manic depressive”] AND [“descendants” or “parent” or “offspring” or “family risk”]) AND (“IQ” or “cognitive functioning” or “neuropsychological” or “Wechsler”) to identify peer-reviewed articles published between 1980 and 2017. This search yielded a total of 136 independent articles. We examined all of the abstracts and read the full text of relevant articles to determine if they met the inclusion/exclusion criteria described below. A total of 11 studies met our review criteria based on this search. Seven additional studies were identified by careful review of the references of relevant articles and literature reviews. In total, 18 studies met inclusion/exclusion criteria for this study. 

We included studies that focused on the offspring of parents diagnosed with a bipolar spectrum disorder or a single type of bipolar disorder (e.g., bipolar I disorder) as determined by diagnostic interview or chart review. Studies were excluded when family risk was defined more broadly, such as offspring of parents with affective disorders, or when the study did not specifically examine BDO or report results for BDO (e.g., [37]). We included studies that used healthy controls or normative data as a comparison group (CO). We only included studies that used validated, standardized measures of IQ. Studies that did not report on core features of IQ such as Full Scale IQ (FSIQ), Verbal IQ (VIQ) and Performance IQ (PIQ)or only assessed specific aspects of neuropsychological functioning (e.g., executive functioning) or achievement testing were excluded, as were studies that specifically matched BDO and control groups on IQ) [23,38]. Some of the studies documented the presence of current or previous offspring psychopathology, but studies were not excluded based on this criterion. When the same group of researchers reported IQ results in multiple publications from what appeared to be largely overlapping samples that were obtained through similar funding lines, every effort was made to determine if there was overlap in participants and accordingly to choose the manuscript with the most inclusive sample of participants of relevance to this review. We allowed for multiple studies from the same research group if they appeared to be conducted with different indexes of IQ and independent samples (only one group met this criteria [39,40]).

## 3. Results

### 3.1. Characteristics

Included in this review were studies conducted in the United States (*n* = 10), Canada, Brazil, United Kingdom, Netherlands, Czech Republic, Spain, Turkey, and China. The studies were generally of low power, with most featuring 20 to 50 BDO (range 15 to 140; median 31). A description of parent’s diagnosis, the offspring characteristics (age, sex), the type of IQ measure, and key demographic characteristics are presented in Table 1. There was considerable variability in the demographic variables reported for each study, but overall the BDO and the CO were similar (with regard to sex and age with the exception of [41] in which age differences were noted across the groups). It was common for the range of ages for offspring to be broad (e.g., such as from 12 to 21 years of age [42]), but on average offspring were in middle to late adolescence. There was no evidence that findings varied systematically by the country in which the study was conducted or by the demographic characteristics of the offspring (e.g., age, sex). 

There was considerable uniformity in the psychiatric instruments used to diagnose parents with BD, typically the Structured Clinical Interview for the DSM (SCID) or the Schedule for Affective Disorders and Schizophrenia (SADS). Diagnostic criteria for BD were quite similar irrespective of the Diagnostic Statistical Manual (DSM) version used, although some minor variation in criteria is present across DSM-III, DSM-IV, DSM-IV-TR, and DSM-5. There was more variability in the range of diagnoses included in the parental bipolar group, with some studies focusing exclusively on offspring of parents with BD-I [39,40,41,43], some focusing primarily but not entirely on parents with BD-I [42,44], and others using more inclusive criteria for bipolar spectrum disorders that typically included parents diagnosed with BD-I and BD-II disorders [45,46,47,48,49,50,51,52,53,54]. Of note, nearly all studies (at least 14) used the Wechsler Intelligence Scales, which are considered to be the gold standard for IQ assessment. Various versions of the scales were administered depending on the age of the offspring (e.g., Wechsler Intelligence Scale for Children [WISC] versus Wechsler Adult Intelligence Scale [WAIS]), and the time allocated to testing (full battery versus the Wechsler Abbreviated Scale of Intelligence [WASI]). Almost all of the studies reviewed used older versions of the Wechsler Scales (with the exception of de la Serna et al., 2016 who reported on the WISC-IV), and focused on broader summary indexes including Full Scale IQ (FSIQ), Verbal IQ (VIQ) and Performance IQ (PIQ) rather than empirically derived factor scores [45,55,56]. One study [42] compared BDO to normative data and not a recruited control group. 

### 3.2. Findings on Full Scale IQ

FSIQ is considered the best estimate of overall intellectual functioning, or a measure of general abilities-*g*. FSIQ was reported in 16 of the 18 studies included in this review. We found little evidence of higher or lower general intelligence among BDO compared to CO, as 12 of the 16 studies that compared the two groups found no significant difference in FSIQ. Wals and colleagues [42] reported that BDO had a significantly higher IQ score than the general population, but no control group was included in their study (they also excluded BDO with IQ lower than 70). It is notable that, of the 16 studies reporting FSIQ, all but three found lower FSIQ in BDO than CO. There were three studies that reported a significantly lower FSIQ in BDO than CO [43,50,52]. One of the studies reported an especially large effect size (*d* = −0.99; [52]). This study differs from the other studies included in the review because the participants in the BDO sample were recruited based on severe family risk that extended BD to additional generations beyond just the parents and because the WASI was administered to controls but the full battery was administered only to BDO. By contrast, the other two studies recruited participants from the community and were therefore more comparable to the other studies we reviewed. Thus, it is possible that there is a very modest decrease in FSIQ among BDO, thought the effect may be an effect that is difficult to detect in the relatively small samples typically included in of offspring studies. It is also possible that deficits in intellectual functioning might be present in some BDO samples based on the severity of the familial loading or other contextual factors. Indeed, the group differences in one study [50], failed to account for the fact that BDO in their sample were of lower socioeconomic status (SES) than CO [31]. On the other hand, Sharma and colleagues [43] found evidence of lower FSIQ on the WASI among BDO compared to CO even after accounting for SES in their mixed effects model and selectively sampling BDO with no significant mental health or neurological problems. Nonetheless, these studies highlight the importance of assessing a range of moderators when studying variation in intellectual functioning among BDO. Another possibility warranting consideration is that deficits in intellectual functioning are isolated within specific subdomains of intelligence. Investigating this possibility requires the use of more nuanced measures of intellectual functioning that can delineate between lower-order intelligence domains, including VIQ and PIQ. 

### 3.3. Findings on Verbal and Performance IQ

Here we provide a brief overview of some of the current evidence for domain-specific deficits in intellectual functioning for BDO. Early on, there was an interest in evaluating VIQ and PIQ as a reflection of hemispheric specialization [59]. While the models of hemispheric specialization have been revised considerably in the last several decades [60], considering behavioral indicators of cognitive functioning across a range of domains continues to be a priority. We identified seven studies reporting on performance and verbal indexes of IQ, four of which reported on VIQ and PIQ [39,43,54,58], two of which reported on estimates of PIQ [44,51] and one of which provided estimates of these scores based on WISC-IV factors [45]. 

Four of the seven studies failed to find significant differences for BDO and CO in VIQ and PIQ [39,44,51,54]. Among the three studies that found a difference between the groups, Decina and colleagues [58] reported both lower PIQ and a greater VIQ > PIQ discrepancy in BDO compared to CO. Sharma and colleagues [43] reported a slightly different pattern, with both lower PIQ and VIQ in BDO. They also found that although there were no group differences for VIQ > PIQ discrepancy (*p* = 0.13), there were substantially more BDO with a 20 point differences between VIQ and PIQ, suggesting that variability was greater in BDO compared to CO. McDonough-Ryan and colleagues [39] also found a greater VIQ > PIQ discrepancy (as measured by a discrepancy evident in 5% of the population) in BDO compared to CO. Finally, a study conducted by de la Serna and colleagues [45] used the Spanish version of the WISC-IV to assess BDO and CO and reported no difference in VIQ-PIQ discrepancy between BDO and CO. However, it is interesting to note that there was some evidence for group differences across the two main skills that were conventionally assessed as part of PIQ, the Perceptual Reasoning Index and the Processing Speed Index. More specifically, BDO showed marginal impairments in performance on the Perceptual Reasoning Index (*p* = 0.10), which taps an individual’s ability to examine a problem, draw upon visual-motor and visual-spatial skills, organize their thoughts, and create a workable solution. Similarly, BDO were significantly worse than CO on the Processing Speed Index (*p* = 0.001), a measure that assesses visual perception and organization, visual scanning, and hand-eye coordination. This latter result was somewhat surprising given that previous studies have not accounted for the possibility that processing speed is impaired in BDO. 

Together, these data suggest that there is no consistent evidence of a VIQ difference between BDO and CO. It remains possible that BDO have slight impairments in PIQ, or specific components of PIQ, when compared to CO. As with FSIQ, all seven studies found that BDO had lower PIQ scores than CO (only a few being statistically significant), suggesting that the small sample sizes may be hiding a very modest effect. Larger sample sizes or use of a meta-analytic technique that could account for the between-study variance could help determine whether this effect is real. 

## 4. Discussion

The aim of this paper was to conduct a narrative review of the literature to determine whether offspring of parents with bipolar disorder showed evidence of impaired or enhanced intellectual functioning. Family risk studies are critically important, primarily for identifying genetic and environmental contributions to the development of bipolar disorder and potential precursors, but also for determining if BDO should be targeted for preventive intervention. Ideally, our more focused review of the literature would limit some of the methodological variability across studies and be able to provide definitive evidence regarding the presence or absence of intellectual deficits in a particular group of youth at familial risk for bipolar disorder. Almost a decade ago, a similar review failed to find evidence of differences between relatives of BD and healthy controls, noting that relatives of BD were well within the average IQ range in all studies [34]. Our aim was to update and extend the findings from this review by including 10 more recently published studies and by using more stringent criteria to limit methodological variability and exclude duplicate data that may compromise the results. Like the previous review, we failed to find consistent evidence of lower or higher intellectual functioning in BDO when IQ was estimated by FSIQ, with BDO scores generally falling within the average to the above average range of functioning relative to normative samples. Evidence from a small number of studies raises the possibility of more intra-individual variability among BDO, with possible impairments in visual-motor problem-solving skills or processing speed. 

Our conclusions are largely consistent with other family risk studies that employed broader inclusion criteria (e.g., studies that include in their risk group a range of first-degree relatives like siblings, parents, and offspring). Using this broader inclusion criteria, there were several studies that failed to find FSIQ differences between patients with bipolar disorder, their first-degree relatives, and healthy controls [61,62,63,64,65,66], with some noted exceptions showing lower FSIQ in relatives of patients with BD (e.g., [67,68]. There were several family risk studies that also found lower PIQ or a larger discrepancy between VIQ and PIQ for those at family risk for BD (e.g., [64,68]. Together, these findings provide additional evidence that those at family risk for BD have intellectual functioning that is largely preserved, but perhaps with greater intra-individual variability. 

When drawing conclusions about intellectual functioning, it is important to reconcile differences between the studies included in this review and the most definitive offspring study to date: a large-scale, high-risk cohort study recently conducted in Australia [69]. Although Morgan and colleagues did not assess IQ per se, they examined rates of intellectual disabilities in offspring of parents with bipolar disorder and healthy controls, primarily based on a classification of those who were receiving services due to performing two standard deviations or more below the population mean on an IQ test and showing significant deficiencies in adaptive functioning. Results of the study indicated that BDO (*n* = 1301) were about three times more likely to be classified as having severely impaired intellectual functioning compared to low-risk offspring in the healthy comparison group (*n* = 3129), but there were no significant differences in the rates of intellectual disability between BDO and the offspring of parents with depression or schizophrenia. Unlike the studies reviewed in Table 2 that considered IQ as a dimensional measure, Morgan and colleagues focused exclusively on the categorical classification of intellectual disability. Thus, they were not able to investigate the range or inter-individual variability of intellectual functioning in BDO. By only focusing on one tail of the distribution, this study could not rule out the possibility that BDO have both lower and higher intellectual functioning than CO, which could theoretically produce high inter-individual variability. Nor can it determine whether the average IQ in BDO is lower than in CO. Nevertheless, the results of this large cohort study limit the strength of the conclusions from this review and suggest the need for more careful analysis of the variability in intellectual functioning among BDO. 

### 4.1. Recommendations for Further Inquiry into Inter- and Intra-Individual Variability

One approach to investigating inter-individual variability in BDO is to examine whether the variance or standard deviations of FSIQ are greater for BDO than CO. If there were both higher and lower IQ performance in BDO, mean IQ might not differ across groups, potentially explaining some of the inconsistencies between Morgan and colleagues’ findings and the conclusions of this review. However, the studies we reviewed (Table 2) do not generally support this hypothesis. Although several studies trend toward more FSIQ variance in BDO than CO [47,50,52,53,58], there are equally as many studies in which CO show more variance in FSIQ than BDO [39,40,41,43,46,48,49]. 

A second index of variability that is only beginning to be explored is deviation from a family cognitive aptitude score, in which IQ and educational attainment of individuals at risk for BD are compared to their predicted IQ and educational attainment that is based on their overall family index of these factors. We did not identify any studies of offspring of parents with bipolar disorder that examined deviation from family cognitive aptitude, although one large study used this method in siblings of a BD-I proband [70]. They found no differences in IQ and school achievement in the siblings of patients diagnosed with BD-I and matched controls, but the risk for BD-I was weakly predicted by deviations from family cognitive aptitude, consistent with findings that either high or low IQ may be predictors of BD.

Inter- and intra-individual variability in IQ can also be assessed by examining narrow domains of intellectual functioning, such as performance or verbal IQ. Unfortunately, many of the studies reviewed here relied on abbreviated assessments of IQ, such as the WASI, and are therefore not ideal for evaluating within-individual variability. Nevertheless, seven studies reported on either VIQ, PIQ, or within-individual differences in VIQ and PIQ scores. Three of the studies found lower PIQ [43,45,58] and two others found VIQ > PIQ in BDO compared to CO [39,58]. In the general population, VIQ > PIQ is a less common pattern of discrepancy than PIQ > VIQ. Although there is evidence that both patterns of discrepancies are likely to be linked with learning problems, there is a weaker link between VIQ > PIQ pattern and low achievement than PIQ > VIQ [71]. Additionally, while it remains less clear if educational achievement is retained in BDO [39,72], there is some evidence that good school performance is a risk factor for BD [73]. Even when those diagnosed with BD have lower IQ than controls, they still have been found to reach a higher educational attainment [74]. 

A final approach to examining variability in intellectual functioning, which has yet to be systematically undertaken with BDO, is to examine subtest variability. Variability in subtests (e.g., verbal intelligence, verbal analogies, mathematical knowledge and nonverbal reasoning) has been associated with challenges in school, neural disconnectivity [75], and an increased risk for psychosis [22,76]. Interestingly, subtest variability has not been associated with non-psychotic bipolar disorder or depression [22]. Overall these results suggest that, despite the absence of evidence showing FSIQ differences between BDO and controls, the assessment of inter-individual and intra-individual variability in IQ warrants more attention in the future.

### 4.2. Recommendations for Continued Inquiry into Potential Moderators 

Inconsistencies in the results of this review may be due to a failure to take relevant moderators into account, such as the presence of psychopathology in offspring or the specific manifestations of BD that parents or offspring present with. It will be important when conducting future research to systematically account for offspring psychopathology. As noted by Olvet et al. [36], it is possible that, when intellectual deficits are identified, they are a result of preexisting mental health challenges in the offspring and not family risk status per se. Indeed, in some of the studies to date that have identified lower IQ in BDO than CO, it is possible that offspring psychopathology partially account for these IQ deficits [50,52]. Nonetheless, a clear picture has yet to emerge from the few studies that have systematically accounted for offspring psychopathology. Contrary to predictions, there is some evidence for lower IQ in unaffected BDO as compared to CO (the finding was marginal in one study that used an unspecified index of IQ [48]; the same finding was significant in another study using the WASI [43]). By contrast, Hanford and colleagues [47] found a trend for the IQ of partially affected BDO (M = 104.4) to be lower than unaffected BDO (M = 114.2) or CO (M = 114.7). Similarly Lin and colleagues [51] showed a trend for lower IQ in BDO compared to CO, but not in unaffected BDO. Finally, some of the studies that accounted for offspring psychopathology found no evidence of IQ differences between affected and unaffected BDO or between unaffected BDO and CO [40,41,42,49]. Most of these studies considered a range of psychopathology in the affected BDO, and not just bipolar disorder. There are other features of psychopathology, aside from symptoms of BD, which also warrant consideration. The developmental course of cognitive impairments may differ for those primarily presenting with manic features compared to those presenting with psychotic features. For example, Murray and colleagues [77] proposed that early insults impair neurodevelopment in individuals with psychotic disorders, but not in patients with BD. Similarly, in a review of the literature, Lewandowski and colleagues [78] concluded that schizophrenia was characterized by premorbid cognitive impairment. In contrast, those with BD exhibited normative cognitive functioning prior to the onset of disorder, but then a deteriorating (and sometimes fluctuating) course of neurocognitive functioning accompanying the onset and exacerbation of symptoms. Thus, it is possible that cognitive functioning in BDO may be impaired in a subgroup of BDO at risk for or exhibiting symptoms of psychosis, but intact in BDO who are not at risk for psychosis and who do not yet display symptoms of bipolar disorder. 

Critical aspects of the symptom profile are thought to influence the way intellectual performance may also vary across risk populations. For example, the presence of sleep disturbance, commonly associated with hypomania and mania, may impair cognitive functioning for those at high risk for and those diagnosed with bipolar disorder (e.g., [79]). Additional evidence suggests that the presence of hypomanic and depressive symptoms in BDO may be related to cognitive impairment overall, despite conferring some limited performance advantages in domains affecting creativity [80]. In other words, performance during assessment may be moderated by the participant’s current symptoms. By employing designs that consider risk patterns associated with a wider range of mood disorders, there is an opportunity to identify both common and unique family risk indicators between bipolar disorder and other affective disorders [69,81]. 

Finally, it is possible that IQ functioning is sexually dimorphic in BDO [82]. Most of the studies that found enhanced IQ in those who went on to develop BD used conscript samples composed of males (e.g., [5,19,25]; an exception is a cohort study [26]). Relatively little is known about how IQ varies in male and female BDO across development or the relationship between IQ, sex, and risk for BD in offspring. In one of the only studies on BDO that reports sex differences, male offspring of parents with BD-I had marginally higher IQ than female offspring [42]. At a minimum, these identified differences in IQ patterns in BDO suggest that researchers should be cautious in assuming the same developmental risk trajectories for girls and boys.

## 5. Limitations

There were several limitations to this review, many of which suggested fruitful avenues of research to pursue in the future. To the best of our knowledge this review includes all non-redundant published studies that evaluate IQ functioning in BDO, but our results are based on the number of studies yielding significant deficits rather than analytic approaches that focus on effect sizes or meta-analysis. Given the fact that most studies employed relatively small samples, a meta-analysis could help resolve some of the inconsistent findings. One possible limitation stems from the risk of publication bias, the tendency for negative findings to go unpublished. While publication bias is always a major concern, the results of this review are less likely to be affected by publication bias because IQ was a primarily outcome in only a minority of studies (most primary outcomes focused on other topics like neural mechanisms). The one finding at high risk for publication bias was VIQ-PIQ discrepancy, given that this pattern was identified early on in the literature but has been infrequently reported in recent studies. A second limitation is that the reviewed studies did not typically correct for multiple comparisons, which increase the likelihood of type I errors. However, we tended to be conservative in drawing conclusions from inconsistent findings, so as not to read too much into the findings from small, often inconsistent, results.

One of the most notable limitations is the small sample size in the majority of studies reviewed (reported in Table 1). Five studies out of 18 had a total sample larger than 100, and only one study had a BDO sample over 100. Thirteen studies had samples with less than 50 BDO. Although these studies still serve an important hypothesis-generating function, their small sample sizes limit the conclusions than can be drawn from them and increase the likelihood of false-positive results. These studies will need to be expanded upon in other larger and potentially more representative samples, as has been occurring recently with large epidemiological and multisite studies investigating other aspects of intellectual functioning in bipolar disorder (e.g., [42,83]. Selection bias may also impact the results. Recruitment approaches for the BDO and CO are typically different. For example, Goetz et al., [44] recruited BDO offspring from a registry and from psychiatric hospitals with psychiatric outpatient services, but recruited CO through advertisements in schools. Additionally, it is not clear to what extent the findings would be generalizable to other BDO. The results may show higher performance than is typical given that the studies reviewed represent parents who have engaged in the mental health community and are able or willing to be evaluated or engage in treatment. These families may also have more resources available to them than the general population. Additionally, it is common to rule out participants with IQs lower than 70 or 80 (e.g., [42,43]). However, researchers do not typically report the number of participants per group excluded based on this criteria so it is unclear the extent to which this selection bias may be affecting IQ estimates in BDO or CO. It is notable that most studies found above average IQ in both BDO and CO (some more than a full standard deviation above average), suggesting that studies were biased in favor of individuals with higher IQ and may not be representative of the general population. It is also possible that studies published later may be mindful of potential IQ differences in their sample as they continue recruitment. For example, some studies have noted IQ differences in early comparisons while not identifying differences in later comparisons despite what seem to be partially overlapping samples (e.g., [84] versus [40]; [85] versus [86] versus [49]). Indeed, there are a number of studies that explicitly matched for IQ in their recruitment approach (e.g., [87]) that we excluded from this review. 

There were a number of limitations regarding the methods and analytic approaches of the studies included in this review. While some preliminary considerations of subgroups were explored in this review, integrative models of multiple family risk factors should be considered in future research that account more fully for the interplay between these nested, interrelated system of parental capacities and resources. Analytic approaches such as mixed models used by Sharma and colleagues [43] provide a useful approach for considering non-independence in the future. 

Indeed, one of the pressing problems for this field to consider further is the overlap between offspring IQ and estimates of family resources, including parental IQ, the severity of parent psychopathology, socioeconomic status, and education. There is compelling evidence of the heritability of IQ and the effect of psychopathology on IQ scores, suggesting that both should be accounted for in studies on the cognitive functioning of BDO [36]. Studies did not typically account for the severity of parent psychopathology or whether parents were receiving treatment, both of which could arguably affect offspring intellectual functioning. Additionally, because SES consistently contributes to a considerable amount of variance in IQ scores, some studies accounted for SES or other relevant indexes such as parental education [39,42,43,45,54,57,58]. Accounting for SES sometimes, but not always, negated group differences between BDO and CO. Furthermore, we have yet to fully appreciate if high or low SES is a risk factor for the onset of BD, for there is some evidence that high SES may increase risk for bipolar disorder [88].

There is also a lack of research on the way in which IQ changes across development in BDO. Studies reviewed here do not provide information about who went on to develop bipolar disorder or other serious psychopathology, making it impossible to say whether the intellectual functioning of BDO could be considered a risk or protective factor for psychopathology. Klimes-Dougan and colleagues conducted follow up evaluations roughly eight years after assessing IQ and found a trend for lower IQ to predict the onset of BD in a small sample of young adults (e.g., [89]). However, other researchers found that low IQ did not predict the development of BD, when risk for BD was based on the presence of prodromal symptoms [90]. Other studies that compare risk for psychotic and affective disorders have found that lower IQ has been shown to predict psychosis but not affective pathology [91]. 

## 6. Conclusions

The results of this review show that intellectual capabilities are largely preserved in offspring of parents diagnosed with BD, although they may demonstrate more intra-individual variability in IQ. These results also are consistent with the mounting evidence that cognitive deficits are not a precursor for BD, but only emerge after the onset of the disorder [78,92]. Future research should more thoroughly investigate inter- and intra-individual variability in intellectual functioning, employ larger samples that are more representative of the general population, and examine the effect of potential moderators of IQ, such as the presence of psychotic symptoms, offspring sex, SES, and parental IQ. This line of work can meaningfully contribute to a deeper understanding of risk and protective factors for bipolar disorders, and can help promote the development and refinement of early interventions for those at risk for BD.

## Figures and Tables

**Table 1:** Studies of Bipolar Offspring-Demographic Characteristics of BDO and CO.

Author	Parental Diagnostic Assessment	Offspring N (Males)		Offspring Age	
		BDO	CO	BDO Mean (SD)	CO
Worland et al. [57]	Interview/Chart Review	26 ^†^ (11 ^†^)	119 (63)	10.2 ^†^	9.2 ^†^
Winters et al. [54]	RDC	76 (32)	134 (69)	6–16 ^a^	NI
Decina et al. [58]	SADS/SADS-L	31 (14)	18 (8)	7–14 ^a^	7–14 ^a^
Wals et al. [42]	IDCL	140 (72)	NI	12–21 ^a^	12-21 ^†^
McDonough-Ryan et al. [39]	SCID-P^BDI^	28 (13)	24 (11)	8–12 ^a^	8–12 ^a^
Frangou et al. [41]	SCID-I (DSM-IV)	15 (5)	43 (24)	27.2 (8.9)	42.9 (11.2)
Klimes-Dougan et al. [50]	SADS-L	43 (17)	50 (26)	15.1 (2.5)	15.3 (2.7)
Maziade et al. [52]	SCID-I (DSM-III-R)	23 (14)	45 (NI.)	17.45 (4.5)	17.32 (4.3)
Versace et al. [53]	SCID-I/II (DSM-IV)	20 (9)	25 (7)	13.2 (2.5)	13.9 (2.6)
Diwadkar et al. [46]	SCID-II (DSM-IV)	23 (13)	41 (25)	14 (2.4)	14.9 (2.7)
De la Serna et al. [45]	SCID (DSM-IV)	90 (50)	107 (48)	12.52 (3.1)	11.71 (3.2)
Gomes et al. [48]	SCID-I (DSM-IV)	17 (11)	24 (8)	14.4 (2.9)	14.8 (2.2)
Hanford et al. [47]	SCID (DSM-IV)	30 (17)	20 (11)	13.4 (2.8)	13.3 (2.5)
Welge et al. [40]	SCID (DSM-IV)	32 (9) ^b^/32 (6) ^c^	32 (11)	15.3 (3) ^b^/14.4 (2) ^c^	14.6 (3)
Goetz et al. [44]	SADS-L	43 (25)	43 (25)	12.5 (3.1)	12.4 (3.1)
Kim et al. [49]	SCID (DSM-III-R)	21 (7) ^b^/24 (14) ^c^	24 (11)	13.4 (2.4) ^b^/13.8 (3.1) ^c^	13.0 (2.4)
Lin et al. [51]	SCID-I/P (DSM-IV-TR)	37 (22) ^b^/21 (12) ^c^	48 (26)	17.2 (5.3) ^b^/14.8 (6.2) ^c^	15.6 (4.5)
Sharma et al. [43]	SCID (DSM-IV)	24 (14)	34 (21)	11.7 (2.6) ^†^	10.2 (2.5) ^†^

Note: BDO, offspring of bipolar parents; CO, offspring of control; RDC, Research Diagnostic Criteria; SADS-L, Schedule for Affective Disorders and Schizophrenia Lifetime version; SADS, Schedule for Affective Disorders and Schizophrenia; IDCL, International Diagnostic Check List; SCID-I (DSM-IV), Structured Clinical Interview for DSM-IV Axis I Disorders; SCID-II (DSM-IV), Structured Clinical Interview for DSM-IV Axis II Disorders; SCID-I (DSM-III-R), Structured Clinical Interview for DSM-III-R Axis I Disorders; SCID-P, Structured Clinical Interview for DSM-IV Axis I Disorders Patient Edition; SCID-NP, Structured Clinical Interview for DSM-IV Axis I Disorders Non-patient Edition. Mo = months; NI–no information. ^†^ = approximately; ^a^ Range; ^b^ unaffected BDO; ^c^ BDO with clinical problems (subthreshold or threshold diagnoses).

**Table 2:** Studies of Bipolar Offspring—Results of IQ.

Author (Year)	Intelligence Assessment	BDO Mean (SD)	CO Mean (SD)	Statistical Significance	Summary of Findings
Worland et al., [57]	WISC				No Differences for FSIQ.
	FSIQ	103.6(NI)	108.9(15.9)	NS	
Winters et al., [54]	WISC				No differences for FSIQ, VIQ and PIQ.
	FSIQ	20.5^@^	20.0^@^	NS^@^	
	VIQ	21.1 (5.1)	20.3 (5.5)	NS	
	PIQ	20.0 (4.9)	20.1 (5.0)	NS	
Decina et al., [58]	WISC-R				No differenes for FSIQ and VIQ. BDO had significantly lower PIQ than CO. The VIQ/PIQ discrepancy was greater for BDO than CO (*t* = 3.30; *p* < 0.005).
	FSIQ	116.2 (13.8)	121.9(7.8)	NS	
	VIQ	118.4 (14.3)	120.5 (9.8)	NS	
	PIQ	110.2 (13.9)	118.6 (9.2)	*t* = 2.26; *p* < 0.05	
Wals et al., [42]	WISC-R/WAIS				BDO had significantly higher FSIQ compared to the general population norms.
	FSIQ	113 (16)	100^@^		
McDonough-Ryan et al., [39]	WISC-III				No differences for FSIQ, VIQ and PIQ. BDO had significantly greater number participants with VIQ > PIQ difference than CO.
	FSIQ	104.9 (13.4)	111.8 (16.2)	*t* = −1.6; *p* = 0.1	
	VIQ	108.7 (17.8)	110.7 (15.5)	*t* = −0.43; *p* = *0.7*	
	PIQ	96.3 (16.6)	105.2 (17.9)	*t* = −1.8; *p* = 0.07	
Frangou et al., [41]	WAIS-R				No differences for FSIQ.
	FSIQ	107 (10.4)^b^	106 (11.0)	NS	
Klimes-Dougan et al., [50]	WISC-R				BDO had significantly lower FSIQ than CO.
	FSIQ	112.5 (15.6)	121.4 (13.7)	*F* = 8.89; *p* < 0.004	
Maziade et al., [52]	WISC/WASI				BDO had significantly lower FSIQ than CO.
	FSIQ	99.6 (12.6)	108.3 (9.4)	*p* = 0.0002 ^a^	
Versace et al., [53]	WISC-III FSIQ	114.7 (13.6)	114.6 (10.1)	*t* = 0.01; *p* = 0.99	No differences for FSIQ.
Diwadkar et al., [46]	Unspecified FSIQ	101.5 (12.9)	101.8 (18.2)	*F* = 0.40, *p* >0.65	No differences for FSIQ.
de la Serna et al., [45]	WISC-IV				No differences for FSIQ (GAI), VCI, and WML. BDO had significantly lower PSI than CO and a trend for lower PRI than CO.
	FSIQ^@^	104.9 (12.9)	107.3 (12.5)	*F* = 1.2, *p* = 0.27	
	VCI	106.1 (13.7)	107.3 (13.8)	*F* = 0.2, *p* = 0 .68	
	PRI	103.9 (14.0)	107.9 (13.8)	*F* = 2.7, *p* = 0.10	
	WMI	100.6 (12.9)	100.7 (14.1)	*F* = 0.2, *p* = 0 .64	
	PSI	98.9 (14.4)	106.7 (12.3)	*F* = 16.4, *p* = 0.001	
Hanford et al., [47]	WASI FSIQ	108.6 (16.3)	114.7 (12.5)	*t =* 1.4, *p* = 0.2	No differences for FSIQ.
Gomes et al., [48]	Unspecified FSIQ	94.5 (9.4) ^b^	100.4 (12.6)	*U* = 3.9, *p* = 0.2	No differences for FSIQ.
Welge et al., [40]	WASI				No differences for FSIQ.
	FSIQ	101 (12) ^b^/101 (11) ^c^	104 (12)	*p* = 0.6	
Goetz et al., [44]	RPM				No differences for PIQ.
	PIQ^@^	115.1 (15.7)	116.5 (14.2)	*p* = 0.7	
Kim et al., [49]	WASI				No differences for FSIQ.
	FSIQ	112.4 (10) ^b^/111.5 (10.8) ^c^	114.1 (13.2)	*F* = 0.3, *p* = 0.7	
Lin et al., [51]	TONI-3				No differences for PIQ.
	PIQ^@^	26.6 (8.5) ^b^/24.7 (8.6) ^c^	29.9 (8.2)	*F* = 2.5, *p* = 0.06	
Sharma et al., [43]	WASI				BDO had significantly lower FSIQ, VIQ and PIQ than CO. BDO had significantly greater number participants with VIQ > PIQ difference than CO.
	FSIQ	98.2(11.7) ^b^	106.9 (16.6)	*p* = 0.001	
	VIQ	89.1 (12.1) ^b^	106.4 (18.4)	*p* = 0.001	
	PIQ	97.9 (12.5)^b^	106.1 (14.8)	*p* = 0.03	

Note: Presented in the order of the year of publication. BDO: offspring of bipolar parents; CO: offspring of control; WISC-R: Wechsler Intelligence Scale for Children Revised Edition; WAIS: Wechsler Adult Intelligence Scale; WAIS-R: Wechsler Adult Intelligence Scale Revised Edition; WISC-III: Wechsler Intelligence Scale for Children Third Edition; WAIS-III: Wechsler Adult Intelligence Scale Third Edition; WISC-IV: Wechsler Intelligence Scale for Children, Fourth Edition; FSIQ: full scale IQ, PIQ: Perceptual IQ, VIQ: Verbal IQ; GAI: Global Abilities Index, VCI: Verbal Comprehension Index; PRI: Perceptual Motor Index; WMI: Working Memory Index; PSI: Processing Speed Index; RPM: Raven’s Progressive Matrices; TONI-3: Test of Non-verbal Intelligence, Third Edition. ^@^ = approximate (in Winters et al., we estimated FSIQ from PIQ and VIQ; in de la Serna et al., we estimated FSIQ from the GAI; in Goetz et al., we used RPM as an estimate of PIQ, in Lin et al., we estimated PIQ from TONI-3); Values in bold represent statistically significant results. ^a^ *p*-value from post hoc analyses using Fisher’s least significant difference and Cohen’s *d*; ^b^ unaffected BDO; ^c^ BDO with clinical problems (subthreshold or threshold diagnoses).
